# Crohn's Disease, Hemochromatosis, Hepatocellular Carcinoma, and Liver Cirrhosis: A Case Study

**DOI:** 10.7759/cureus.67781

**Published:** 2024-08-26

**Authors:** Dalia Alrawashdeh, Noim Jibon, Ali Raheem, Saravanaa Sankar, Vinod Warrier

**Affiliations:** 1 Internal Medicine, Southend University Hospital NHS Foundation Trust, Southend-on-Sea, GBR; 2 Internal Medicine, Mid and South Essex NHS Foundation Trust, Southend-on-Sea, GBR

**Keywords:** venesection, liver transplant, hepatocellular carcinoma (hcc), multi-disciplinary teams, secondary iron overload, crohn’s disease (cd), hereditary haemochromatosis, live cirrhosis

## Abstract

Hemochromatosis, an inherited disorder characterized by excessive iron absorption and accumulation, can lead to organ damage and is a known contributor to liver cirrhosis. This case report discusses a 57-year-old man with a history of Crohn's disease, whose general practitioner identified elevated ferritin levels, cirrhotic liver features, and abnormal liver function tests. Further investigation revealed non-hereditary hemochromatosis, hepatic cirrhosis, and hepatocellular carcinoma (HCC).

This case highlights the rare coexistence of hemochromatosis and Crohn's disease, underscoring the diagnostic and therapeutic challenges of managing these concurrent conditions. It also emphasizes the importance of prompt and effective treatment to prevent severe complications.

## Introduction

Hemochromatosis is an iron storage and overload disorder that can be either primary (genetic) or secondary (acquired) [1]. Primary hemochromatosis involves 20 known mutations of the HFE gene, with C282Y and H63D being the most common [2]. Non-HFE hemochromatosis is less frequent and includes hepcidin-deficient hemochromatosis, such as hemojuvelin (HJV type 2A), hepcidin (HAMP type 2B), and TRF2-related hemochromatosis (type 3) [3]. Other forms include ferroportin disease (type 4A) and atypical ferroportin disease (type 4B), with the V162del mutation reported in non-C282Y hemochromatosis [3].

Type 1 hemochromatosis, also known as classic hereditary hemochromatosis, is caused by mutations in the HFE gene, most commonly C282Y and H63D. This is the most common type of hemochromatosis and typically manifests in adulthood, leading to excessive iron absorption and accumulation in organs such as the liver, heart, and pancreas. Type 2 hemochromatosis, or juvenile hemochromatosis, results from mutations in the HJV (Hemojuvelin) gene (Type 2A) or the HAMP (Hepcidin) gene (Type 2B). This form of the disease manifests earlier, usually in adolescence, and is characterized by a rapid progression with severe iron overload and complications, including cardiac issues and endocrine dysfunctions. Type 3 hemochromatosis is associated with mutations in the TFR2 (Transferrin Receptor 2) gene and has an intermediate onset, typically in young adulthood. The symptoms and iron overload in this type are similar to those of Type 1 but generally occur at a younger age. Type 4 hemochromatosis, also known as ferroportin disease, is linked to mutations in the SLC40A1 (Ferroportin) gene. This form of the disease usually presents in adulthood with iron accumulation primarily in macrophages, leading to different patterns of organ involvement and iron loading.

Hemochromatosis results in excess dietary iron absorption from the intestinal tract, leading to iron deposition in tissues, particularly the liver, heart, pancreas, pituitary gland, joints, and skin. Clinically significant tissue iron deposition generally occurs after several decades of excess iron absorption without concomitant blood loss, usually by the fifth decade of life or later. Common clinical findings include fatigue and arthralgia, with other features such as hyperpigmentation, loss of libido, and hyperglycemia. Phlebotomy is the mainstay of treatment for hemochromatosis [3].

Crohn’s disease (CD) is one of the two major types of inflammatory bowel disease (IBD) and is more prevalent in developed countries. It typically presents in early adulthood with symptoms like abdominal pain, diarrhea, hematochezia, and weight loss. Extraintestinal manifestations can include arthritis, uveitis, ankylosing spondylitis, and erythema nodosum. Symptoms vary widely depending on the severity and location of inflammation. Several factors, including environmental influences, autoimmunity, genetics, and gut microbiome derangement, contribute to the development of CD [4]. Complications can include fistulas, abscesses, obstruction, and internal bleeding. Patients with CD are also at higher risk for other autoimmune conditions such as primary sclerosing cholangitis, celiac disease, type 1 diabetes, sarcoidosis, psoriasis, rheumatoid arthritis, and ankylosing spondylitis [5].

## Case presentation

A 57-year-old male presented with symptoms including abdominal pain, night sweats, weight loss, fatigue, and sexual dysfunction. Physical examination revealed bronze pigmentation on the forehead, submandibular region, and shins, suggestive of hemosiderin deposition. The patient had a background of hypertension and CD since 1999, for which he was not under any treatment. Elevated ferritin levels (1827 ng/mL) noted in 2019 suggested a history of iron overload, potentially indicative of underlying hemochromatosis, although this was not fully investigated at the time.

Blood tests revealed deranged liver function, including elevated alkaline phosphatase (ALP), alanine transaminase (ALT), total bilirubin, and gamma-glutamyl transferase (gamma GT), indicating liver injury and impaired liver function. The presence of iron overload was suggested by elevated serum ferritin levels, transferrin saturation, and the patient's clinical presentation, prompting further investigation into the possibility of hemochromatosis (Table 1). His blood tests showed serum ferritin at 520 ng/mL, transferrin at 1.16 g/L, and transferrin saturation at 71%.

**Table 1:** Laboratory tests on the first day of hospital admission. ALP: alkaline phosphatase, ALT: alanine aminotransferase, PT: prothrombin time, APTT: activated partial thromboplastin time, INR: international normalized ratio, HB: hemoglobin, WCC: white cell count.

Description	Results	Normal values
ALP	181 U/L	30–130 U/L
Total bilirubin	88 µmol/L	0–21 µmol/L
ALT	29 U/L	<50 U/L
Albumin	26 g/L	35–50 g/L
Globulin	51 g/L	20–35 g/L
PT	20.2 seconds	10.3–13.3 seconds
APTT	37.6 seconds	25.7–35.3 seconds
INR	1.6	0.8–1.2
HB	131 g/L	115–165 g/L
WCC	5.8 × 10^9^/L	4.0–11.0 × 10^9^/L
Platelet	97 × 10^9^/L	150–400 × 10^9^/L

**Figure 1:**
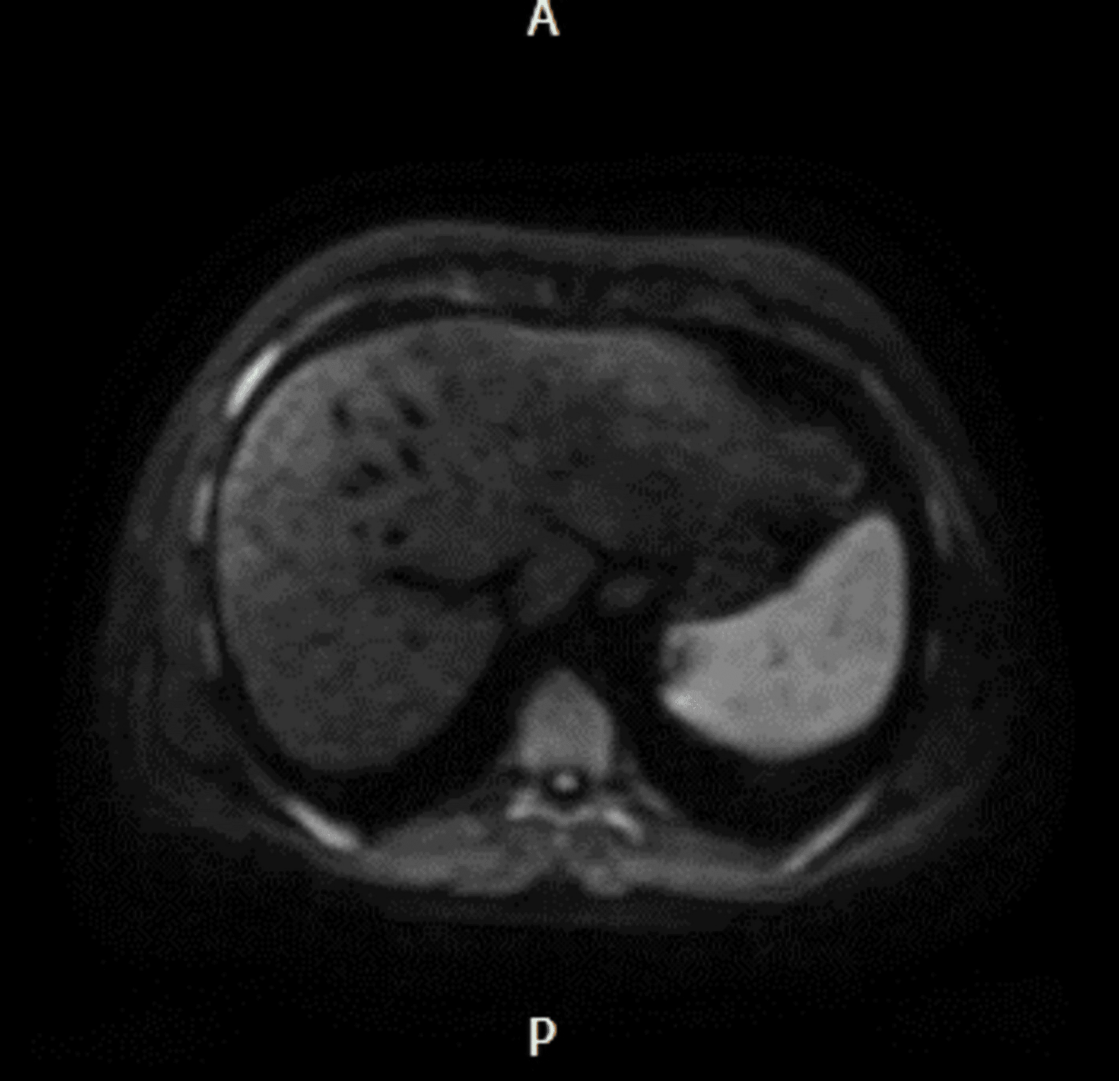
MRI showing an enlarged spleen and multiple enhancing lesions in the liver.

Imaging studies, including ultrasound and MRI, confirmed the presence of advanced liver disease with cirrhosis, portal hypertension, and ascites. Liver nodules identified on imaging were consistent with hepatocellular carcinoma (HCC), a common complication of cirrhosis (Figure 1).

Gastroscopy demonstrated oesophageal varices, a consequence of portal hypertension, increasing the risk of potentially life-threatening variceal bleeding. A genetic study was sent for hemochromatosis.

The patient was diagnosed with cirrhosis with portal hypertension and ascites, indicative of advanced liver disease (Child-Pugh Class C). This was complicated by HCC, which was classified as LR 5. Genetic testing for hemochromatosis revealed heterozygosity for the H63D mutation.

Management involved a multidisciplinary approach, including hepatology, oncology, and transplant teams. This approach focused on surveillance and management of complications such as variceal bleeding, ascites, and hepatic encephalopathy, with consideration for liver transplantation.

He was started on carvedilol for the management of oesophageal varices to reduce the risk of variceal bleeding. Venesection was not initiated due to decreased transferrin levels, indicating no immediate need for iron reduction therapy. A liver transplant was successfully performed in January 2024.

Post-transplant, the patient is being continuously monitored for complications such as graft rejection, infection, and recurrence of HCC. The long-term prognosis after liver transplantation is favorable, provided there is no significant extra-hepatic damage. His iron overload is being carefully monitored to prevent complications. He is in regular follow-up with hepatology, oncology, and transplant teams to ensure optimal post-transplant recovery and function.

## Discussion

Hemochromatosis is a genetic disorder characterized by excessive iron absorption and deposition in various organs, leading to systemic iron overload. The pathophysiology of hemochromatosis primarily involves a deficiency or dysfunction of hepcidin, the key hormone regulating iron homeostasis. Hepcidin inhibits the cellular efflux of iron by binding to and inducing the degradation of ferroportin, the sole iron exporter in iron-transporting cells. In hemochromatosis, reduced hepcidin activity or impaired hepcidin-ferroportin interaction results in uncontrolled iron absorption and accumulation [4].

The most common form of hereditary hemochromatosis is linked to mutations in the HFE gene, particularly the C282Y mutation [4]. Individuals homozygous for the C282Y mutation typically present with significant iron overload and are at risk for developing complications such as cirrhosis, diabetes mellitus, and cardiomyopathy. Other mutations, such as H63D in HFE or mutations in non-HFE genes like HAMP, HJV, TFR2, and SLC40A1 (FPN1), also contribute to iron overload but tend to have a variable clinical impact. These mutations disrupt normal iron sensing and regulation, leading to progressive iron accumulation in organs like the liver, heart, and pancreas [4].

Diagnosis of hemochromatosis is based on clinical evaluation, laboratory findings (elevated serum ferritin and transferrin saturation), imaging studies, and genetic testing [4]. Early detection is crucial to prevent irreversible organ damage. Imaging techniques such as MRI can quantify liver iron content and detect complications like cirrhosis and hepatocellular carcinoma (HCC). Genetic testing confirms the diagnosis and helps identify at-risk family members.

The primary treatment for hemochromatosis is phlebotomy, which effectively reduces iron stores by removing blood at regular intervals [4]. This simple and cost-effective treatment can prevent or mitigate complications if initiated early. In patients who cannot tolerate phlebotomy or have secondary iron overload, iron chelation therapy may be used.

CD is a chronic IBD with a relapsing-remitting course, affecting any part of the gastrointestinal tract. Its etiology is multifactorial, involving genetic susceptibility, environmental triggers, and immune dysregulation. One intriguing aspect of CD is its potential genetic overlap with hemochromatosis. Both conditions are associated with the HLA region on chromosome 6, which includes the HFE gene [3]. Although the precise relationship between these diseases is not fully understood, shared genetic factors might contribute to their co-occurrence in some patients.

In CD, chronic inflammation and frequent gastrointestinal bleeding can lead to iron deficiency anemia [3]. However, the presence of hemochromatosis can complicate the clinical picture, as iron supplementation for anemia must be carefully managed to avoid exacerbating iron overload. Additionally, chronic liver disease in Crohn's patients can be exacerbated by concurrent hemochromatosis, increasing the risk of cirrhosis and HCC [3].

Management of patients with coexisting hemochromatosis and CD requires a multidisciplinary approach [3]. Coordination among gastroenterologists, hepatologists, and hematologists is essential to balance the treatment of iron overload and the management of inflammatory bowel disease. Regular monitoring of iron parameters and liver function tests is vital to adjust therapy appropriately and prevent complications [3].

## Conclusions

In summary, this case highlights the uncommon co-occurrence of hemochromatosis and CD in a single patient, underscoring the importance of early diagnosis to enable the implementation of an appropriate treatment plan, which can ultimately prevent complications. Our patient underwent a liver transplant for hepatocellular carcinoma and is experiencing a favorable prognosis. He is being monitored for iron levels and post-transplant medications. Hemochromatosis is a significant genetic disorder with potential systemic effects, especially when associated with other conditions like CD. A thorough understanding of the pathophysiology, genetic factors, and clinical management of hemochromatosis is essential for improving patient outcomes. Early diagnosis, timely therapeutic interventions, and a multidisciplinary approach are critical to effectively managing this complex disorder. Additionally, patients with heterozygous H63D mutations are at an increased risk of developing HCC, regardless of the underlying cause of liver cirrhosis.
